# Balloon aortic valvuloplasty for urgent treatment of severe aortic stenosis during coronavirus disease 2019 pandemic: a case report

**DOI:** 10.1002/ehf2.13003

**Published:** 2020-09-19

**Authors:** Tiziana Attisano, Angelo Silverio, Michele Bellino, Carlo Tumscitz, Fabio Felice Tarantino, Andrea Santarelli, Cesare Baldi, Rodolfo Citro, Gennaro Galasso

**Affiliations:** ^1^ Interventional Cardiology Unit, Cardiovascular and Thoracic Department University Hospital San Giovanni di Dio e Ruggi d'Aragona Salerno Italy; ^2^ Department of Cardiology University Hospital of Ferrara Ferrara Italy; ^3^ Cath Lab Unit, Cardiovascular Department Morgagni Hospital Vecchiazzano‐Forlì Italy; ^4^ Interventional Cath Lab Unit, Cardiovascular Department Infermi Hospital Rimini Italy

**Keywords:** Balloon aortic valvuloplasty, Transcatheter aortic valve implantation, Aortic stenosis, Novel coronavirus, COVID‐19, SARS‐CoV‐2, Case report

## Abstract

An 86‐year‐old man affected by severe aortic stenosis (AS) was referred to our institution owing to decompensated heart failure. Three months before, the patient was scheduled for transcatheter aortic valve implantation (TAVI), which was postponed owing to the coronavirus disease 2019 (COVID‐19) outbreak. Owing to COVID‐19 suspicion, he underwent nasopharyngeal swab and was temporarily isolated. However, the rapid deterioration of clinical and haemodynamic conditions prompted us to perform balloon aortic valvuloplasty (BAV) as bridge to TAVI. The patient's haemodynamics improved; and the next day, the reverse transcriptase–polymerase chain reaction for COVID‐19 was negative. At Day 5, he underwent TAVI procedure. Subsequent clinical course was uneventful. During COVID‐19 pandemic, the deferral of TAVI procedure should be assessed on a case‐by‐case basis to avoid delay in patients at high risk for adverse events. BAV may be an option when TAVI is temporarily contraindicated such as in AS patients suspected for COVID‐19.

## Introduction

During the last decade, transcatheter aortic valve implantation (TAVI) has made a paradigm shift in the treatment of severe symptomatic aortic stenosis (AS) not suitable for surgical replacement. More recently, it has been proposed as an option also in patients with low‐to‐intermediate surgical risk.^1^ Therefore, the number of patients candidate to this procedure has grown considerably, demanding considerable economic and human resources for scheduling patients and perform procedures.^2^

The majority of TAVI procedures are performed on an elective basis and, therefore, have been postponed during coronavirus disease 2019 (COVID‐19) outbreak. However, delay in the treatment of severe AS has been associated with the risk of recurrent hospitalization and mortality during wait‐time, particularly in higher‐risk patients with older age and multiple co‐morbidities.^3^, ^4^

In this scenario, a medical treatment combined with balloon aortic valvuloplasty (BAV) may represent a viable therapeutic option, particularly in patients with suspected or confirmed severe acute respiratory syndrome coronavirus 2 (SARS‐CoV‐2).

We describe, for the first time, the case of a patient with severe AS, already scheduled for TAVI at our institution and admitted for congestive heart failure refractory to medical therapy. Owing to the suspicion of SARS‐CoV‐2 infection, we decided to perform a BAV as a bridge to TAVI pending the result of the nasopharyngeal swab virologic test.

## Case

An 86‐year‐old man affected by severe AS was referred to our institution from a peripheral centre owing to decompensated heart failure refractory to optimal medical therapy.

The patient's medical history was remarkable for atrial fibrillation under treatment with non‐vitamin K oral anticoagulants, diabetes, peripheral artery disease, and severe chronic obstructive pulmonary disease.

**Figure 1:**
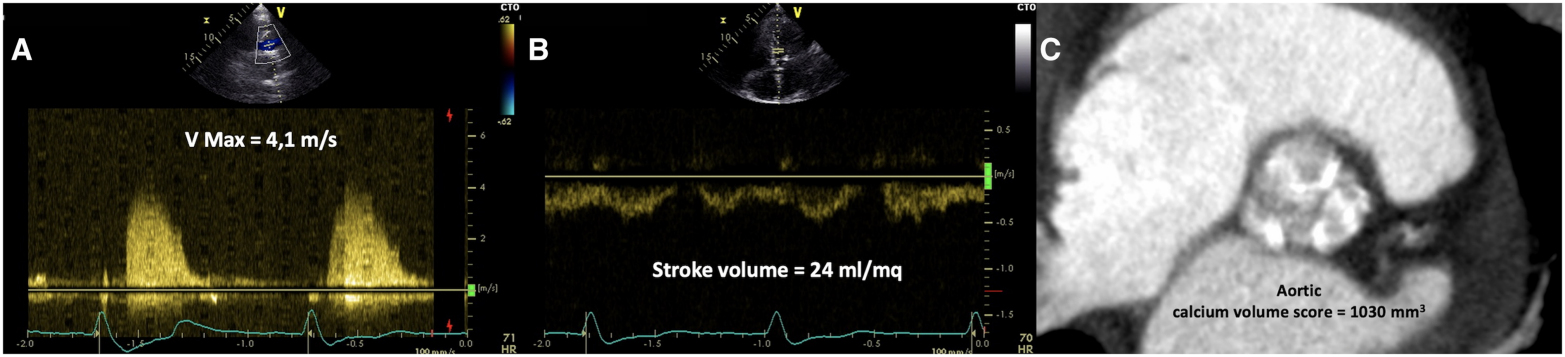
(A) TTE continuous wave Doppler on right parasternal view showing peak aortic velocity of 4.1 m/s. (B) TTE pulsed‐wave Doppler on left ventricle outflow tract evidencing an indexed stroke volume of 24 mL/m^2^. (C) Contrast‐enhanced multi‐slice computed tomography short‐axis view showing the aortic valve characterized by a high calcium volume (1030 mm^3^). TTE, transthoracic echocardiography.

Three months before, the patient was evaluated by our Heart Team and was scheduled for TAVI. Transthoracic echocardiography (TTE) showed a severe AS (indexed aortic valve area = 0.25 cm^2^/m^2^; mean gradient = 42 mmHg; *Figure* *1*) associated with severe left ventricular (LV) systolic dysfunction (LV ejection fraction = 30%). At contrast‐enhanced multi‐slice computed tomography (CT), the aortic annulus area was 4.9 cm^2^ (annulus perimeter = 8 cm) with a high calcium volume (>1000 mm^3^; *Figure* *1*). Since that time, he had been waiting for planned admission and elective TAVI procedure, which was postponed owing to the COVID‐19 outbreak.

**Figure 2:**
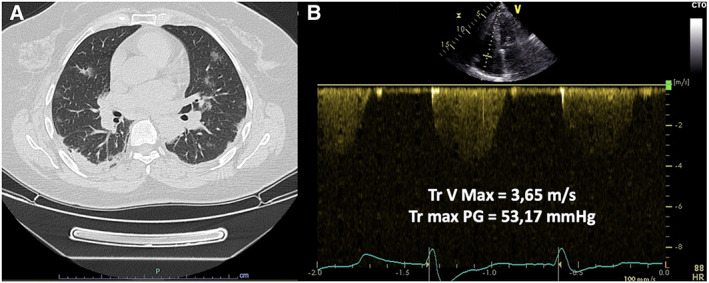
(A) TTE continuous wave Doppler on tricuspid valve suggestive for severe pulmonary hypertension. (B) Chest computed tomography revealed bilateral ground‐glass opacifications and smooth interlobular and intralobular septal thickening suggestive for interstitial pneumonia. PG, peak gradient; TTE, transthoracic echocardiography.

Before being transferred to our hospital, the patient developed fever, chills, and cough; therefore, he underwent chest CT, which revealed bilateral ground‐glass opacifications, smooth interlobular and intralobular septal thickening suggestive for interstitial pneumonia, and thin right pleural effusion hilar congestion (*Figure* *2*). Owing to the high suspicion of SARS‐CoV‐2 infection associated with clinical and CT signs of congestive heart failure, nasopharyngeal swab was rapidly obtained. At arrival in our centre, the patient was isolated in the COVID unit, as a precautionary measure. Systolic blood pressure was 90 mmHg, and electrocardiogram showed high‐rate atrial fibrillation (mean heart rate 120 b.p.m.). Laboratory exams revealed leucopenia (white cell counts, 4200 U/mm^3^), high serum levels of C­reactive protein (50 pg/mL; normal value < 3 mg/mL) and of brain natriuretic peptide (BNP = 3724 pg/mL; normal value < 35 pg/mL). TTE confirmed the severity of the AS and of the LV systolic dysfunction (indexed stroke volume = 24 mL/m^2^) associated with severe tricuspid regurgitation and pulmonary hypertension (systolic pulmonary arterial pressure = 53.2 mmHg; *Figure* *2*). Owing to the rapid deterioration of clinical and haemodynamic conditions (blood arterial saturation was 85% and systolic blood pressure down to 90 mmHg), despite pharmacological and non‐invasive ventilatory supportive measures, the patient was referred to the catheterization laboratory to be considered for rescue percutaneous treatment of the AS. We decided to perform a BAV owing to the patient's unstable conditions and the suspicion of SARS‐CoV‐2 infection (results of nasopharyngeal swab for COVID‐19 still pending), and to postpone any decision for TAVI.

The procedure was executed in compliance with the doffing and donning protocol and within the rules of personal protective equipment, as recommended.^5^ BAV was preceded by coronary angiography, which revealed the absence of critical coronary lesions. The procedure was performed via the right femoral artery by positioning of an 8 French sheath.

After aortic valve crossing through a long exchange guidewire, a manually J‐shaped super‐stiff guidewire (INNOWI® 0.035 TAVI WIRE; SYMEDRIX GmbH, Deisenhofen, Deutschland) was placed into the LV apex through a pigtail diagnostic catheter.

**Figure 3:**
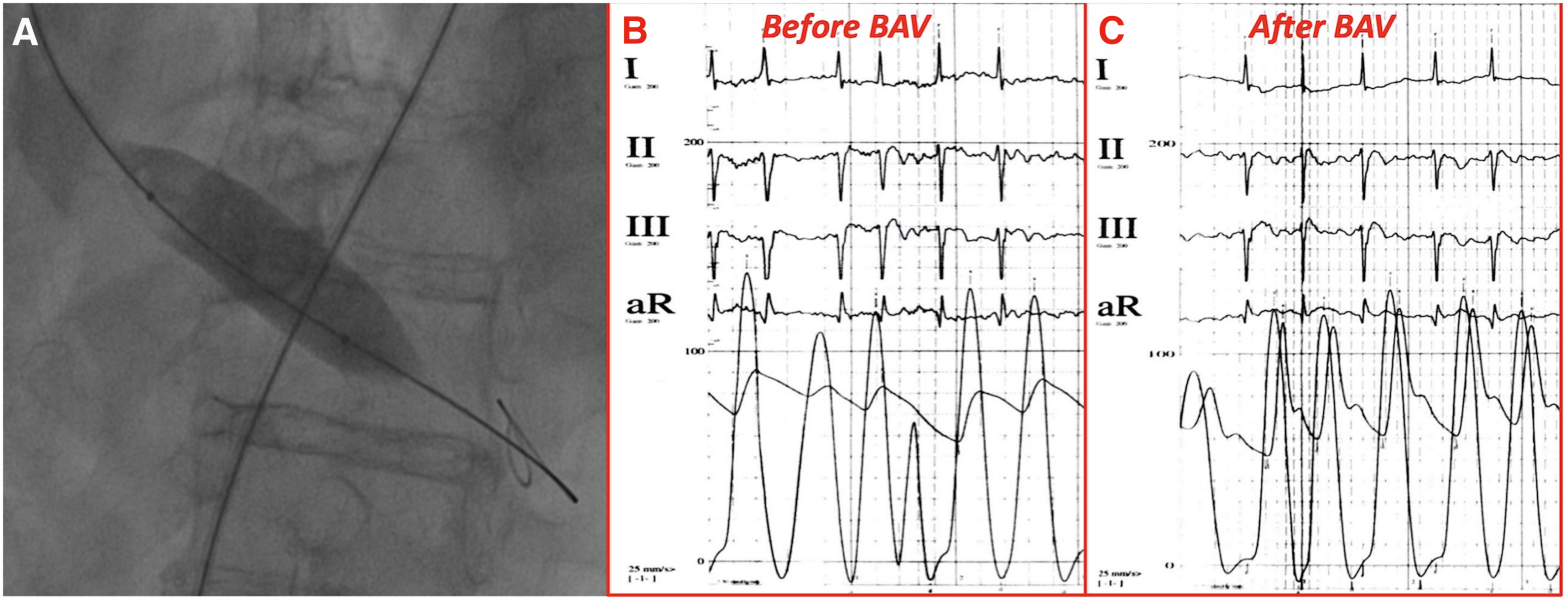
(A) Fluoroscopic image of balloon inflation phase of BAV during high‐rate LV pacing; invasive transaortic pressure gradient before (B) and after (C) balloon aortic valvuloplasty showing a significant reduction of gradient from 43 to 10 mmHg. BAV, balloon aortic valvuloplasty; LV, left ventricular.

During temporary high‐rate (180 b.p.m.) LV pacing through the retrograde LV support wire (positive electrode at the groin, negative on the wire), a Cristal Balloon 20 × 45 mm (Balt, Montmorency, France) was inflated (*Figure* *3*). A reduction of the invasive transaortic gradient from 43 to 10 mmHg was achieved after one inflation (*Figure* *3*); and no complications were observed. After procedure, the patient was transferred in the COVID unit and closely monitored, waiting for result of nasopharyngeal swab. Patient clinical conditions markedly improved, and systolic blood pressure increased to 120 mmHg in few hours.

TTE confirmed the reduction of the transvalvular aortic gradient associated with improved LV systolic function (ejection fraction = 40%; indexed stroke volume = 32 mL/m^2^) and lower systolic pulmonary arterial pressure (31 mmHg**)**. BNP was markedly reduced (751 pg/mL).

The next day, the result of the reverse transcriptase–polymerase chain reaction was negative. In view of the high suspicion of COVID‐19, nasopharyngeal swab was repeated and confirmed as negative. The patient was apyretic and clinically and haemodynamic stable in the next days.

At Day 5, he underwent TAVI procedure; and CoreValve Evolut™ Pro 29 (Medtronic, Dublin, Ireland) was successfully implanted. Subsequent clinical course was uneventful. He was discharged at Day 8 in good clinical conditions.

## Discussion

The key messages of this clinical case can be summarized as follows:

- Although TAVI is generally performed on an elective basis, the deferral of the procedure in the context of COVID‐19 pandemic should be assessed on a case‐by‐case basis, in order to avoid delay in patients at high risk for adverse events.
- BAV may be an option in heart failure patients with severe AS when TAVI is temporarily contraindicated such as in patients suspected for COVID‐19.

Although TAVI is generally performed on an eligible basis, there is still a need to figure out how to best manage the list for TAVI as well as to develop benchmarks for the maximum acceptable waiting time for patients with severe AS pending intervention.^6^ During COVID‐19 outbreak, we experienced a substantial reduction in patient admission in emergency room for any pathology. The whole scientific community is very concerned about this phenomenon, as it could lead to an increase of mortality as an indirect effect of the pandemic. Patients with severe AS represent a high‐risk group for developing adverse events at short term and mid‐term, mostly if affected by multiple co‐morbidities, and they may not tolerate long waiting time for intervention.^7^

Owing to the constrained resources of health care system and the risk of infection during COVID pandemic, this patient, already scheduled for TAVI, paid the price for waiting too long and was admitted in a peripheral centre for the acute onset of congestive heart failure refractory to medical therapy.

Special consideration deserves the candidacy to percutaneous heart valve procedures in patients with suspected COVID‐19. The risk of in‐hospital infection is clearly an issue of interest in COVID‐19 outbreak for both patients and clinicians. Percutaneous procedures need special attention because the paucity of interventionist cardiologists would make impossible to replace them in case of infection.^8^ Therefore, pandemic mitigation strategies include the reduction of face‐to‐face clinical contacts and unnecessary eligible interventions that may expose health care professionals to risk of infection and consume protective equipment.^9^, ^10^

The presence or suspicion of SARS‐CoV‐2 infection should not preclude a systematic search for acute cardiovascular events such as decompensated heart failure. Although both diseases can present dyspnoea and chest CT abnormalities, the medical history of AS and the high BNP serum level were strongly suggestive for heart failure reacutization in our patients. Moreover, the rapidly deteriorating haemodynamic conditions and the TTE evidence of low cardiac output associated with pulmonary hypertension supported the cardiac aetiology of that condition.

The haemodynamic instability and resistance to optimized drug therapy required in this patient an emergency interventional treatment. However, the potential benefit of TAVI needed to be weighed against the periprocedural risks and the likelihood of futility. Previous observational studies, in fact, emphasized the highest rate of mortality in elderly subjects with COVID‐19 as well as in those with multiple co‐morbidities.^11^, ^12^

Therefore, we did not perform TAVI at first; but waiting for results of nasopharyngeal swab, we preferred to perform BAV to get the patient out of the haemodynamic instability. The shorter procedure time of BAV, compared with TAVI, reduced the operators' exposure to the potential contagion as well as the need of anaesthesiologist's support.

BAV is a safe and effective procedure that can be performed by trained operators in centres not performing TAVI without transferring to another centre, reducing potential viral spread.^13^ Moreover, the risk of vascular and bleeding complications may be further limited by performing mini‐invasive BAV via radial artery. This recent approach may allow faster mobilization after procedure and could be preferred in higher‐risk patients.^14^ Radial access, considered the first‐line approach in our centre, was not viable in this case owing to the absence of arterial pulse.

In the COVID‐19 era, the Heart Teams should be very careful in stratifying the risk of patients with severe AS candidates for TAVI and in setting the correct time for intervention. In this patient with suspected SARS‐CoV‐2 infection, BAV had been a valuable option to solve the haemodynamic and allow subsequent TAVI.

## Conclusions

The COVID‐19 pandemic resulted in a reduction or suspension of TAVI procedures and increased the risk of adverse events during the waiting time. BAV may work as a bridging strategy for subsequent TAVI in selected patients affected by severe AS and with suspected SARS‐CoV‐2 infection.

## Consent

The authors confirm that written consent for submission and publication of this case report including images and associated text has been obtained from the patient in line with COPE guidance.

## Conflict of Interest

The authors have no conflict of interest to declare.

## Funding

The authors have no financial support to declare.
